# Association of Cognitive Reserve Indicator with Cognitive Decline and Structural Brain Differences in Middle and Older Age: Findings from the UK Biobank

**DOI:** 10.14283/jpad.2024.54

**Published:** 2024-03-19

**Authors:** W. Yang, J. Wang, J. Guo, A. Dove, Xiuying Qi, D. A. Bennett, Weili Xu

**Affiliations:** 1 Department of Epidemiology and Biostatistics, School of Public Health, Tianjin Medical University, Qixiangtai Road 22, Heping District, 300070 Tianjin, P.R. China; 2 Department of Epidemiology, College of Preventive Medicine, Army Medical University (Third Military Medical University), Chongqing, China; 3 Department of Nutrition and Health, China Agricultural University, Beijing, China; 4 Aging Research Center, Department of Neurobiology, Care Sciences and Society, Karolinska Institutet, Tomtebodavägen 18A Floor 10, Solna, SE-171 65 Stockholm, Sweden; 5 Rush Alzheimer’s Disease Center, Rush University Medical Center, Chicago, Illinois USA

**Keywords:** Cognitive function, cognitive reserve, latent class analysis, magnetic resonance imaging, UK Biobank

## Abstract

**Background:**

Cognitive reserve (CR) contributes to preserving cognition when facing brain aging and damage. CR has been linked to dementia risk in late life. However, the association between CR and cognitive changes and brain imaging measures, especially in midlife, is unclear.

**Objective:**

We aimed to explore the association of CR with cognitive decline and structural brain differences in middle and older age.

**Design:**

This longitudinal study was from the UK Biobank project where participants completed baseline surveys between 2006 to 2010 and were followed (mean follow-up: 9 years).

**Setting:**

A population-based study.

**Participants:**

A total of 42,301 dementia-free participants aged 40–70 were followed-up to detect cognitive changes. A subsample (n=34,041) underwent brain magnetic resonance imaging scans.

**Measurements:**

We used latent class analysis to generate a CR indicator (categorized as high, moderate, and low) based on education, occupation, and multiple cognitively stimulating activities. Cognitive tests for global and domain-specific cognition were administrated at baseline and follow-up. Total brain, white matter, grey matter, hippocampal, and white matter hyperintensity volumes (TBV, WMV, GMV, HV, and WMHV) were assessed at the follow-up examination. Data were analyzed using mixed-effects models and analysis of covariance.

**Results:**

At baseline, 16,032 (37.9%), 10,709 (25.3%), and 15,560 (36.8%) participants had low, moderate, and high levels of CR, respectively. Compared with low CR, high CR was associated with slower declines in global cognition (β [95% confidence interval]: 0.10 [0.08, 0.11]), prospective memory (0.10 [0.06, 0.15]), fluid intelligence (0.07 [0.04, 0.10]), and reaction time (0.04 [0.02, 0.06]). Participants with high CR had lower TBV, WMV, GMV, and WMHV, but higher HV when controlling for global cognition (corrected P <0.01 for all). The significant relationships between CR and cognition and TBV were present among both middle-aged (<60 years) and older (≥60 years) participants. The CR-cognition association remained significant despite reductions in brain structural properties.

**Conclusions:**

Higher CR is associated with slower cognitive decline, higher HV, and lower microvascular burden, especially in middle age. Individuals with high CR could tolerate smaller brain volumes while maintaining cognition. The benefit of CR for cognition is independent of structural brain differences. Our findings highlight the contribution of enhancing CR to helping compensate for neuroimaging alterations and ultimately prevent cognitive decline.

**Electronic Supplementary Material:**

Supplementary material is available in the online version of this article at 10.14283/jpad.2024.54.

## Introduction

There is vast individual variability in the trajectories of cognitive aging and susceptibility to age-related brain changes. A proposed explanation for this is the cognitive reserve (CR) hypothesis, which contends that relevant lifetime exposures enhancing CR can foster more adaptability in cognitive processing, providing a buffer against age-related brain damage (1, 2). However, CR, as a latent construct, cannot be assessed directly, and its quantification and measurement have been a challenge.

For practical purposes, CR is commonly defined in terms of proxy indicators based on socio-behavioral variables. Education level is the most widely used proxy measure of CR, and arguably the easiest to measure. Nevertheless, the measure with a single factor seems incomplete and could not fully reflect CR influenced by various experiences (3, 4). Occupational status and engagement in cognitively-loaded activities have been considered as two other key proxy measures of CR recently (5). Similarly, evidence from epidemiological studies has highlighted the benefits of leisure activity (6) and minimal engagement in mentally passive sedentary behavior, such as television viewing (7), for cognitive health. As such, a comprehensive measure of CR should combine multiple proxy measures to consider a wider range of contributions to CR.

In CR-related research, three components are required including a measure of CR (e.g., a proxy), cognitive outcomes, and a measure of brain lesion burden (1). Several previous studies have linked composite indicators of CR to better cognitive functioning in older age (8–11). Magnetic resonance imaging (MRI) scans are widely applied to measure structural alterations in the brain, where differences in regional brain volumes may reflect the traits of vascular neuropathology and neurodegeneration. However, literature on the association between CR and brain structural measures is limited due to the cost of MRI and has shown inconsistent findings. Among cognitively healthy older adults, some studies have suggested that high levels of CR-related factors are associated with larger brain volumes (12–15), while others reported that high CR is related to more brain atrophy (16–18) and still others indicated no significant association (19, 20). So far, open questions remain about whether CR is associated with brain MRI markers and whether the association between CR and cognition varies by brain MRI markers. Moreover, older adults compared with young ones tend to have faster cognitive decline and worse brain health (21, 22). Studies addressing the above associations have focused mostly on older people. However, few studies have so far examined whether CR may also influence cognition and brain status among younger adults.

We hypothesized that a high level of CR is related to slower cognitive decline in both older (≥60) and middle (40–60) age, independent of brain MRI measures. In the current study, we aimed to verify this hypothesis by examining the association between an indicator of lifelong CR and cognitive decline and exploring the relationship between CR and structural brain MRI measures in middle and older age using data from the UK Biobank.

## Methods

### Study population

The UK Biobank is a community-based prospective cohort study comprising over half a million participants aged 37–73 years recruited from 22 sites across the UK. Between 2006 and 2010, 502,412 individuals participated in the baseline survey and underwent sociodemographic, physical, cognitive, and medical assessments. Between 2014 and 2020 (approximately 9 years later), a subsample (n=48,188) of the population participated in the follow-up examination (including repeated cognitive tests).

**Figure 1:**
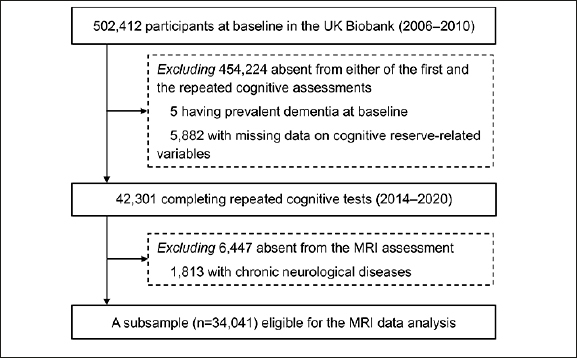
Flowchart of the study population Abbreviations: MRI, magnetic resonance imaging

Of those 48,188 participants, we excluded 5 with prevalent dementia and 5882 with missing data on CR-related variables, leaving 42,301 dementia-free participants for the analysis of the association between CR and cognitive decline. Next, we additionally excluded 6447 who did not undergo MRI assessments and 1813 with chronic neurological diseases (including Parkinson’s disease, brain cancer, brain hemorrhage, brain abscess, aneurysm, cerebral palsy, encephalitis, head injury, nervous system infection, head or neurological injury, and stroke; see Appendix 1 for more details), leaving 34,041 participants for the analysis of the association between CR and structural brain MRI measures (Figure 1).

The UK Biobank study received ethical approval from the North West Multi-Centre Research Ethics Committee (21/NW/0157). All enrolled participants provided informed and written consent, and all data used in this study were obtained from the UK Biobank (http://www.ukbiobank.ac.uk) through application 67048.

### Data collection

At baseline, information on age, sex, ethnicity, smoking, alcohol consumption, and physical activity was self-reported through computerized touch-screen questionnaires.

Ethnicity was dichotomized as white vs. non-white. Cigarette smoking status was grouped as never, previous, or current smoking. Alcohol consumption was categorized as never, previous, or current drinking. Physical activity was measured in terms of metabolic equivalents (MET) per week using the International Physical Activity Questionnaire short form and classified as low (<600 MET-min/week), moderate (600 to <3000 MET-min/week), and high (≥3000 MET-min/week) (23). Body mass index (BMI; kg/m^2^) was calculated as weight (kg) divided by the square of height (m). Heart disease was ascertained through medical records and self-reported medical history. Diabetes was identified based on hemoglobin A1c ≥6.5%, fasting plasma glucose ≥126 mg/dl, self-reported history of diabetes, use of glucose-lowering medications, or medical records. Hypertension was identified based on systolic blood pressure ≥140 mm Hg, diastolic blood pressure ≥90 mm Hg, self-reported history of hypertension, use of antihypertensive drugs, or medical records (see Appendix 1 for details on the ascertainment of heart disease, diabetes, and hypertension). The apolipoprotein E (APOE) gene was genotyped and dichotomized as carriers vs. non-carriers of the ε4 allele. Missing values for smoking status (0.2%), alcohol consumption (0.1%), physical activity (12.8%), BMI (0.1%), and APOE ε4 carrier status (15.8%) were assigned a separate category.

Information on the following CR-related factors was collected at baseline based on self-report: education level, occupation, TV viewing time, frequency of confiding, frequency of social connection, and number of leisure activities.

Education. Education was operationalized as the number of years of formal schooling (24, 25) and was classified as 1) no educational qualifications, Certificate of Secondary Education or equivalent, or O levels/General Certificate of Secondary Education or equivalent, 2) A/AS levels or equivalent, or other professional qualifications, 3) National Vocational Qualification, Higher National Diploma, Higher National Certificate or equivalent, or 4) college/university degree.

Occupation. Participants self-reported their employment status and job titles. Each job title was matched to a job code derived from the Standard Occupational Classification 2000 system which was developed by the UK Office of National Statistics [26]. A simplified socio-economic classification (SEC) was directly derivable from a four-digit job code and thus, SEC was an occupationally-based classification and reflected the conditions of occupations. SEC is coded as ordinal variables ranging from 1.1, 1.2, and 2 to 7, with lower values reflecting greater occupational attainment (i.e., jobs requiring higher skill levels and more training) (27, 28). Since about one third of participants were retired at baseline with unavailable job codes, additional data at follow-up on codes of the historical longest-held job were used. Occupational attainment was categorized into four levels: 1) unemployed, looking after home and/or family, unable to work because of sickness or disability, or SEC 5–7, 2) SEC 4 or SEC 3, 3) SEC 2, or 4) SEC 1.2 or SEC 1.1.

Television (TV). TV viewing time (in hours/day) was assessed based on self-reported total hours of daily TV watching and quartiled as 1) ≥4, 2) 3–3.9, 3) 2–2.9, or 4) <2.

Confiding. Frequency of confiding was assessed based on participants’ responses to a question about how often they were able to confide in acquaintances and defined as 1) never, 2) less than about once a month, 3) about once a week, 4) 2–4 times a week, or 5) almost daily

Social connection. Frequency of social connection was assessed based on participants’ responses to a question about how often they made or received friend/family visits and classified as 1) less than about once a month, 2) about once a week, or 3) more than twice a week.

Leisure activity. Leisure activities included involvement in a sports club or gym, pub or social club, religious group, adult education class, or other group activity. Participants were asked to indicate which activities they engaged in once a week or more often. The level of leisure activity was defined as the number of leisure activities engaged in per week and classified as 1) 0, 2) 1, or 3) 2–5.

All categories with <10% of the sample in the above six variables had been merged with the closest category to yield more informative ones (29), as classified above.

### Generation of CR indicator

A CR indicator was created using latent class analysis (LCA) based on education level, occupational attainment, TV viewing time, frequency of confiding, frequency of social connection, and number of leisure activities. LCA is a well-validated statistical approach that uses mixture modeling to find the optimal fitting model for a set of data. This allows for the identification of hidden clusters by grouping multiple observed variables into a latent variable with mutually exclusive latent classes [30]. We identified a three-latent-class model after comprehensively considering statistics regarding model selection and the uncertainty of posterior classification (Bayesian information criterion 5612.19, mean posterior probabilities=0.78).

Latent class 1 was characterized by high levels of CR-related factors in general. Specifically, there were more favorable levels of education, occupational attainment, and TV viewing time. Latent class 2 represented moderate levels of CR-related factors. In this class, frequency of confiding in others and social connection and the number of leisure activities were relatively higher. Latent class 3 was characterized by less favorable levels of all CR-related factors (Supplementary Table 1). More detailed descriptions of the LCA and the six proxy measures of CR are available in Appendix 1.

### Assessment of cognitive function

Cognitive function at baseline and follow-up was evaluated via five tests administered through a touchscreen interface, including numeric memory (the longest numeric string correctly recalled), prospective memory (successfully carrying out an instruction after a filled delay), pairs matching (the number of errors when recalling positions of matching cards), fluid intelligence (the number of correct answers to logic/reasoning-type questions), and reaction time (mean time to correctly match cards). More details on the cognitive function tests can be found at https://biobank.ctsu.ox.ac.uk/ crystal/label.cgi?id=100026. For the numeric memory and fluid intelligence tests, higher raw scores indicate better cognitive performance. For the pairs matching and reaction time tests, higher raw scores indicate worse cognitive performance, so values were reversed for ease of comprehension. Prospective memory performance was scored as 2 for a correct response on the first attempt, 1 for a correct response on the second attempt, and 0 otherwise. A natural log transformation+1 (ln(x+1)) was applied for the pairs matching test in which scores were zero-inflated and positively skewed. Reaction time was natural-log-transformed to correct a positively skewed distribution. These values from all cognitive tests were individually converted to z-scores, and these were further averaged to yield a measure of global cognition, with higher values reflecting better global cognitive function.

### MRI acquisition and processing

The procedure for brain MRI acquisition and processing of imaging-derived phenotypes (IDPs) in the UK Biobank has been described in detail previously (31, 32). Briefly, participants were scanned with a Siemens Skyra 3T scanner with a standard Siemens 32-channel head coil. Tl-weighted imaging (resolution: 1.0x1.0x1.0 mm; field-of-view: 208x256x256 matrix) and T2 FLAIR imaging (resolution: 1.05x1.0x1.0 mm; field-of-view: 192x256x256 matrix) were performed to provide volumes of brain tissues and structures. Summary measures of brain structure were generated by an image-processing pipeline developed and run on behalf of the UK Biobank, using publicly available image processing tools including the FMRIB Software Library (FSL) version 5.0.10 and FreeSurfer version 6.0 (33).

In the current study, MRI measures included total brain volume (TBV), white matter volume (WMV), grey matter volume (GMV), hippocampal volume (HV), and white matter hyperintensity volume (WMHV), with the unit in mm^3^. Values for WMHV were natural-log-transformed due to a positively skewed distribution. Extreme outlying data points (further than ±4 standard deviations [SD] from the mean) were excluded (0.002% of the total IDP data analyzed).

### Statistical analysis

All analyses were performed using SAS 9.4 (SAS institute, Cary, NC) and STATA SE 15.0 (StataCorp, College Station, TX, USA). Baseline characteristics of the study population were compared according to CR level, and differences among the CR groups were tested using Chi-square tests for categorical variables and one-way analysis of variance followed by pairwise comparison with Bonferroni correction for continuous variables.

Linear mixed-effects models were used to examine the association of CR level (low CR as reference) with changes in global and domain-specific cognitive function. The fixed effect included the CR indicator, follow-up time, and their interaction. The random effect included random intercept and slope, allowing individual differences to be reflected at baseline and across follow-up. All analyses were adjusted for age, sex, ethnicity, smoking status, alcohol consumption, physical activity level, BMI, heart disease, diabetes, hypertension, and APOE ε4 carrier status in multi-adjusted models. In MRI data analysis, analysis of covariance models were used to examine the differences in the least-squares mean (LSM) of brain structural measures between CR levels, additionally adjusting for head size, head position (in terms of x-, y-, and z-axis position coordinates), table position, and baseline levels of global cognitive function. A post-hoc analysis tested pairwise differences between CR levels using Dunnett’s correction (reference: low CR). In addition, we performed stratified analyses according to age at baseline (middle-aged [<60 years] and older [≥60 years]).

To further explore the role of structural brain differences in the association between CR and global cognitive changes, we additionally adjusted for TBV, WMV, GMV, HV, and WMHV in linear mixed-effects models, respectively. These regional brain volumes were further dichotomized as smaller vs. larger volume categories based on their medians corrected for age, sex, and head size. Smaller TBV, WMV, GMV, and HV as well as larger WMHV indicated relatively worse brain structural properties. Interactions between CR level and volume category for each brain MRI measure on global cognitive decline were tested, and models were then stratified by brain volume category to aid the interpretation of the interaction.

In the supplementary analysis, we repeated the analyses in linear mixed-effects models 1) in the MRI subsample (n=34,041), 2) after excluding participants who developed dementia during the follow-up (n=495) to reduce reverse causality, 3) further adjusting for the Townsend deprivation index, a variable reflecting neighborhood-level socio-economic status, and 4) stratifying by sex to examine potential sex differences in the CR-cognition association. Statistical tests were two-tailed and P-values <0.05 were considered statistically significant.

## Results

### Characteristics of the study population

At baseline, the mean age of the 42,301 participants was 54.38+7.43 years (range: 40 to 70 years), and 51.8% were female. Of all participants, 16,032 (37.9%) had low CR, 10,709 (25.3%) had moderate CR, and 15,560 (36.8%) had high CR. Compared with those with moderate or high CR, participants with low CR were more likely to be white, current/former smokers, and non-drinkers and to have high levels of physical activity, higher BMI, and a history of heart disease/diabetes/hypertension. In addition, those with low CR tended to have worse cognitive performances on global cognition, numeric memory, prospective memory, pairs matching, fluid intelligence, and reaction time tests. The proportion of APOE ε4 carriers did not differ significantly between the three CR groups (Table 1). The distribution of baseline characteristics according to CR level was similar among the subsample with MRI data (Supplementary Table 2).

**Table 1:** Baseline characteristics of the study population by cognitive reserve level (n=42,301)

**Characteristics**	**Cognitive reserve**			**P**
	**Low (n=16032)**	**Moderate (n=10709)**	**High (n=15560)**	
Age	54.71±7.42	54.97±7.48	53.63±7.35	<0.001
Female	8586 (53.6)	6533 (61.0)	6770 (43.5)	<0.001
White ethnicity	15155 (94.5)	9904 (92.5)	14015 (90.1)	<0.001
Cigarette smoking				<0.001
Never	8929 (55.9)	6789 (63.5)	10043 (64.6)	
Previous	5798 (36.3)	3300 (30.9)	4679 (30.1)	
Current	1261 (7.9)	602 (5.6)	828 (5.3)	
Alcohol consumption				<0.001
Never	431 (2.7)	239 (2.2)	346 (2.2)	
Previous	407 (2.5)	190 (1.8)	306 (2.0)	
Current	15187 (94.8)	10279 (96.0)	14906 (95.8)	
Physical activity				<0.001
Low	2539 (18.9)	1329 (14.1)	2962 (21.1)	
Moderate	6409 (47.8)	5411 (57.4)	8207 (58.5)	
High	4471 (33.3)	2682 (28.5)	2866 (20.4)	
Body mass index (kg/m^2^)	27.25±4.46	26.31±4.19	26.20±4.13	<0.001
Heart disease	602 (3.8)	235 (2.2)	361 (2.3)	<0.001
Diabetes	552 (3.4)	237 (2.2)	384 (2.5)	<0.001
Hypertension	4071 (25.4)	2264 (21.1)	3190 (20.5)	<0.001
Apolipoprotein E ε4 carriers	3677 (27.6)	2497 (27.4)	3671 (27.9)	0.745
Global cognitive function	0.02±0.67	0.13±0.65	0.19±0.66	<0.001
Numeric memory	−0.02±0.97	0.15±0.91	0.27±0.91	<0.001
Prospective memory	0.02±0.92	0.13±0.72	0.12±0.75	<0.001
Pairs matching	−0.08±0.98	0.00±0.99	0.01±1.02	<0.001
Fluid intelligence	−0.35±0.91	0.18±0.95	0.29±0.98	<0.001
Reaction time	0.19±0.99	0.24±0.95	0.34±0.93	<0.001

Data are presented as means ± standard deviations or number (proportion, %). Missing data: 72 for cigarette smoking; 10 for alcohol consumption; 54 for body mass index; 5425 for physical activity; and 6709 for apolipoprotein E ε4 status.

### Association between CR and cognitive decline

**Figure 2:**
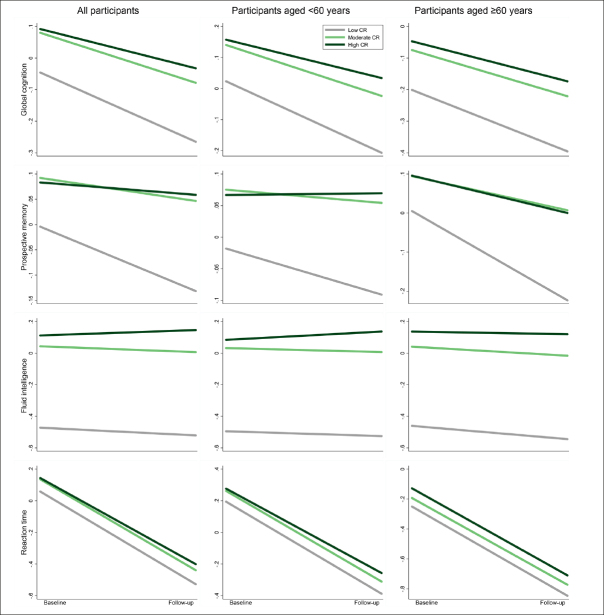
Cognitive trajectories in global cognition and different domains by cognitive reserve (CR) level: results from linear mixed-effects models The trajectories represent β coefficients adjusted for age, sex, ethnicity, cigarette smoking, alcohol consumption, physical activity, body mass index, heart disease, diabetes, hypertension, and apolipoprotein E ε4.

Over the follow-up period (median 9.30 years, interquartile range 7.64 to 10.44 years), compared with low CR, moderate/high CR was associated with slower declines in global cognition (β 0.06, 95% confidence interval [CI]: 0.04 to 0.08 / β 0.10, 95% CI: 0.08 to 0.11) and prospective memory (β 0.08, 95% CI: 0.03 to 0.13 / β 0.10, 95% CI: 0.06 to 0.15) in multi-adjusted mixed-effects models. These associations were significant among middle-aged as well as older participants. In addition, high CR was associated with a slower decline in fluid intelligence and reaction time, especially in middle age. No significant association between CR and numeric memory or pairs matching was detected (Figure 2 and Supplementary Table 3). In stratified analysis by APOE genotype, the CR-cognitive decline association was similar in both ε4 carriers and non-carriers (Supplementary Table 4).

### Association between CR and structural brain differences

**Figure 3:**
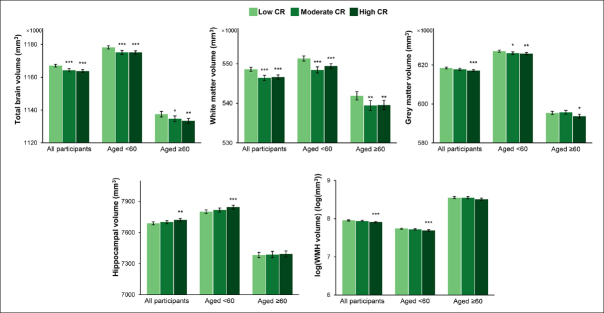
Brain structural magnetic resonance imaging parameters by cognitive reserve (CR) level: results from analysis of covariance models The bars represent the values of least-squares means (95% confidence intervals) of regional brain volumes adjusted for age, sex, ethnicity, cigarette smoking, alcohol consumption, physical activity, body mass index, heart disease, diabetes, apolipoprotein E ε4, head size, position magnetic resonance imaging confounds, and baseline level of global cognitive function. Asterisks indicate a significant difference compared with the low CR group with Dunnett’s correction (*** P <0.001; ** P <0.01; * P <0.05). Abbreviations: WMH, white matter hyperintensity

The median of the intervals between the baseline survey and the MRI scan was 9.17 (range 4.23 to 12.85) years. In analysis of covariance models, participants with moderate or high compared with low CR had smaller TBV (LSM difference: −2795, 95% CI: −4240 to −1351 for moderate CR; LSM difference: −3222, 95% CI: −4553 to −1892 for high CR) and WMV (LSM difference: −2152, 95% CI: −3101 to −1203 for moderate CR; LSM difference: −1943, 95% CI: −2817 to −1068 for high CR) along with relatively preserved cognitive function. Furthermore, those with high CR had smaller GMV (LSM difference: −1280, 95% CI: −2034 to −525) and WHMV (LSM difference: −0.05, 95% CI: −0.07 to −0.02) as well as larger HV (LSM difference: 35, 95% CI: 12 to 57). In age-stratified analyses, the associations of high CR with TBV, WMV, and GMV remained significant in both age groups, while the associations of high CR with HV and WMHV were only significant among middle-aged participants (Figure 3 and Supplementary Table 5).

### Role of structural brain differences in the CR-cognition association

**Figure 4:**
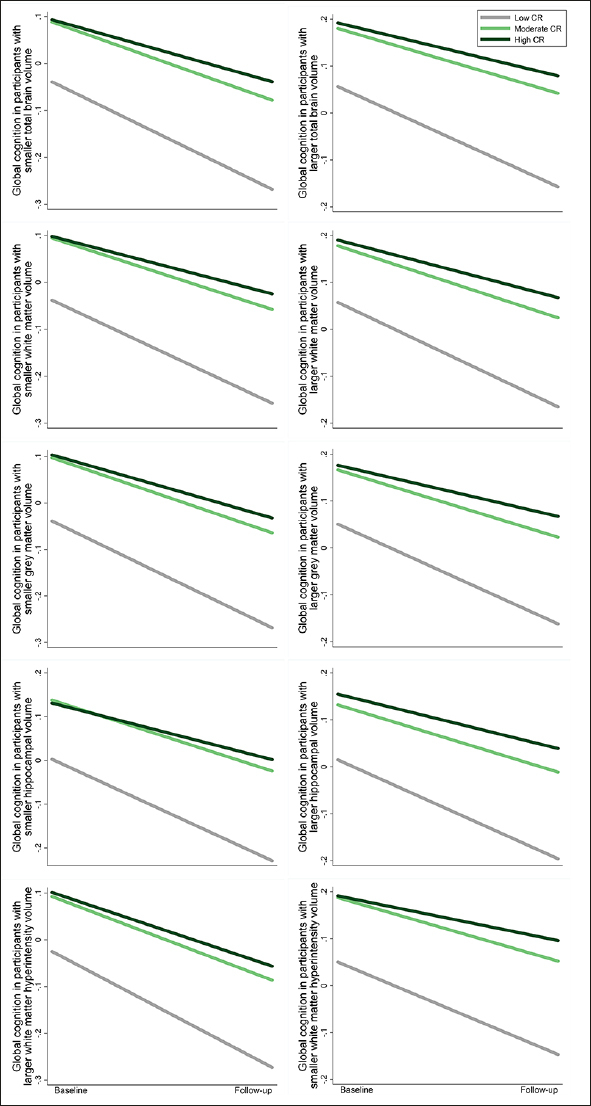
Cognitive trajectories in global cognition by cognitive reserve (CR) level, stratified by brain volume category: results from linear mixed-effects models The trajectories represent β coefficients adjusted for age, sex, ethnicity, cigarette smoking, alcohol consumption, physical activity, body mass index, heart disease, diabetes, hypertension, and apolipoprotein E ε4.

The results on the association between CR and global cognitive function were not substantially altered after additional adjustment for regional brain volumes (Supplementary Table 6). Furthermore, there was no significant interaction between CR and regional brain volumes on global cognition (P >0.05 for all). The effects of moderate and high CR on slower declines in global cognition remained significant among participants with different levels of regional brain volumes (Figure 4 and Supplementary Table 7). These results suggest that the association between CR and global cognitive changes is independent of structural brain MRI measures.

### Supplementary analysis

The results were not much altered when we repeated the analysis of CR-cognition association after 1) restricting the study population to just the MRI subsample (Supplementary Table 8), 2) excluding incident dementia cases (Supplementary Table 9), and 3) additionally adjusting for the Townsend deprivation index (Supplementary Table 10). In addition, the association between CR and cognitive decline remained significant in both male and female participants (Supplementary Table 11).

## Discussion

In this large community-based cohort from the UK Biobank, using a comprehensive CR indicator constructed from multiple socio-behavioral proxies, we found that 1) high CR was associated with slower declines in global cognitive function and prospective memory across middle and older ages, and with slower declines in fluid intelligence and reaction time in middle age; 2) high CR was related to smaller TBV but greater HV and less microvascular burden, especially in middle age; and 3) the association between high CR and slower global cognitive decline was independent of structural brain differences.

Accumulating evidence suggests that individual sociobehavioral measures including higher education (34), higher occupational attainment (35), less time spent watching TV (36), and more frequent social interaction (37, 38) are related to better cognitive function. Individually, however, these single components may not fully reflect the CR construct which can be influenced by a range of experiences in life. So far, several studies have evaluated the relationship between CR and changes in cognitive function using a composite index of CR (8, 11, 39). Two of them have shown that higher CR (indexed by education, reading, and vocabulary measures (11) or education and occupation level (39)) is associated with better cognitive performance but not a change in cognition during the follow-up. In our previous work, higher CR (indexed by education, cognitive activity, and social activity) might predict slower declines in global cognitive function, episodic memory, and working memory in older adults (8). The present study used LCA to construct a comprehensive CR indicator, including education level, cognitively stimulating activities, and previously unmeasured proxies like occupational attainment and screen-based sedentary behavior. We also took the further step of evaluating the association between CR and cognition in both middle and older ages. We found that high CR was associated with a slower decline in global cognition, prospective memory, fluid intelligence, and reaction time, especially in middle-aged adults. This suggests that the beneficial effect of CR on some specific cognitive functions may be stronger in midlife and weaken in later life. Additionally, there was no significant sex difference in the observed associations. Our findings underscore the contribution of CR to the maintenance of cognitive function in both women and men, especially earlier in the life course.

In the field of CR, three necessary components should be taken into account: measures of CR, cognitive function, and the burden of brain lesions (1, 40). Neuroimaging techniques have an excellent ability to estimate structural alterations in the brain, thereby allowing the exploration of possible mechanisms underlying the changes in cognition related to CR. Different brain structural changes could reflect different pathological processes. For instance, a reduction in WMV may be indicative of vascular neuropathology (41, 42), whereas a reduction in GMV and HV could reflect neurodegeneration (43). Furthermore, high WMH burden may suggest microvascular lesions in the brain (41).

So far, limited studies have addressed the association between CR and structural neuroimaging measures in dementia-free individuals, and with inconsistent results. Some studies have suggested a positive correlation between the level of CR-related factors and TBV (12, 15), GMV (13, 15, 44), and HV (45), while others have shown that individuals with higher education have lower cortical thickness and greater age-related brain atrophy (16, 17, 46), even for young adults. In addition, another literature has reported a decreased GMV in cognitively healthy older persons with high CR (indexed by education and cognitive activity) (18). The discrepancies could be due to differences in the age of the study population, assessment of CR-related factors, and inclusion or exclusion of participants with neurological diseases. Additionally, many of these previous studies were limited by relatively small sample sizes ranging from 16 to 348. In the current study including brain MRI data from over 30,000 dementia- and neurological disease-free participants, we found that high CR was related to smaller TBV, WMV, and GMV in both middle-aged and older adults, controlling for cognitive level. This did not necessarily imply, however, that high CR could lead to reductions in brain volumes. Instead, our findings of greater brain atrophy in conjunction with relatively well-preserved cognitive functioning for those possessing high CR are consistent with the reserve hypothesis, which suggests that individuals with higher lifelong engagement in various reserve-enhancing activities can tolerate or cope with more age-related brain changes without exhibiting cognitive impairment (1, 47). Thus, CR might play a neuro-compensatory role. Indeed, in our study, the association between high CR and preserved cognitive function remained significant in the presence of reductions in brain structural properties and was independent of brain MRI measures.

These results further suggest that the mechanism underlying the association between CR and cognition might not fully relate to structural brain changes but rather other pathways. For example, high CR might confer more efficient cognitive processing strategies, including the use of brain networks and the ability to call up alternate networks, which is beneficial to cognitive performance (2). High CR may also contribute to greater neuronal density and neurotrophic changes in the prefrontal lobe (48), which helps compensate for or cope with brain aging. On the other hand, consistent with earlier studies (45, 49, 50), we also found that high CR was related to a greater HV and less WMH burden in middle age. This suggests that high CR might have a neuroprotective effect in some brain regions; while with brain aging, there might be a continuous transition in the role of CR from neuroprotective to neuro-compensatory (51). Further population-based brain imaging studies with longitudinal assessments and different neuroimaging measures beyond structural MRI (such as the measurement and characterization of functional brain processes, thought as the closest direct measure of CR (1)) are warranted to better understand the relationship between CR and brain MRI measures.

The main strength of this study lies in the use of LCA to develop a composite CR indicator incorporating multiple CR-related factors with a good overall fit. Additionally, information on structural brain differences from MRI scans has been taken into consideration in this study, allowing us to assess possible mechanistic explanations for the association between CR and cognition. Nevertheless, the following limitations should be acknowledged. First, since the brain MRI scans were conducted about 9 years after baseline, we could not consider baseline levels of MRI measures and could not evaluate the longitudinal association between CR and long-term changes in brain structure. Second, because the assessment of cognitive trajectories did not occur after the MRI scans, it is challenging to fully examine whether CR can modulate the effect of structural brain differences on subsequent cognitive changes. However, the model as we conducted is also sufficient; it examines the association between the CR indicator and cognition after partialing out the role of brain changes and the association between the CR indicator and brain measures when controlling for cognitive level (1, 47). Third, we could not take into account a non-linear cognitive trajectory over the follow-up, as cognitive function was assessed at two time points. Fourth, the cognitive tests administered in the UK Biobank are brief and non-standard in nature, and some may have low reliability. Despite this, improvement in reliability has been shown when these cognitive tests are combined (52), hence our focus on global as well as domain-specific cognitive function. Finally, the UK Biobank population is predominantly white and highly educated, so our findings should be generalized to other populations with caution.

In conclusion, our study shows that higher CR is associated with slower declines in global and some domain-specific cognitive functions in both middle and older ages. Individuals with high CR are able to withstand or cope with smaller brain volumes without observable deficits in cognition. The protective role of CR on cognition is independent of structural brain differences. Our findings highlight that enhancing CR may help the acquisition of abilities to compensate for neuroimaging alterations and ultimately help in the prevention of cognitive decline. Further large population-based longitudinal studies are required to investigate the mechanisms underlying the association between CR and cognitive health.

### Electronic Supplementary Material

Supplementary material, approximately 80.2 KB.

## Data Availability

*Data Availability:* Access to UK Biobank data can be requested through a standard data access procedure. Requests to access these datasets should be directed to http://www.ukbiobank.ac.uk/register-apply.

## Notes

*Ethical Standards:* The authors assert that all procedures contributing to this work comply with the ethical standards of the relevant national and institutional committees on human experimentation and with the Helsinki Declaration of 1975, as revised in 2008.
