# Supplementary material for: Association of Cognitive Reserve Indicator with Cognitive Decline and Structural Brain Differences in Middle and Older Age: Findings from the UK Biobank
Source: J Prev Alzheimers Dis. 2024 Mar 19;11(3):739–48. doi: 10.14283/jpad.2024.54 (PMC11061039; doi:10.14283/jpad.2024.54)
Supplement: Supplementary file 1 — Supplementary material, approximately 80.2 KB. [file 42414_2024_54_MOESM1_ESM.docx]

**Supplementary material**

**Appendix 1**

**Supplementary Method 1: Ascertainment of chronic neurological diseases**

Because the associations we focused on might be driven by prevalent neurological disorders that affect brain function, participants with such conditions were excluded from the MRI analyses. Chronic neurological diseases in the current study included Parkinson’s disease, dementia or Alzheimer’s disease, brain cancer, brain hemorrhage, brain abscess, aneurysm, cerebral palsy, encephalitis, head injury, nervous system infection, head or neurological injury, and stroke. Information on these conditions was collected by self-report. The UK Biobank field codes for these variables are listed in the following table.

**Table 1 for supplementary method.** Self-reported health variable codes for chronic neurological diseases.

| **Self-reported illness** | **Code (Field ID 20002 and 20003)** |
| --- | --- |
| Parkinson’s disease | 1262 |
| Dementia or Alzheimer’s disease | 1263 |
| Chronic degenerative neurological | 1258 |
| Guillain-Barré syndrome | 1256 |
| Multiple Sclerosis | 1261 |
| Other demyelinating disease | 1397 |
| Stroke or ischemic stroke | 1081 |
| Brain cancer | 1032 |
| Brain hemorrhage | 1491 |
| Brain/intracranial abscess | 1245 |
| Cerebral aneurysm | 1425 |
| Cerebral palsy | 1433 |
| Encephalitis | 1246 |
| Epilepsy | 1264 |
| Head injury | 1266 |
| Infections of the nervous system | 1244 |
| Ischemic stroke | 1583 |
| Meningeal cancer | 1031 |
| Meningioma (benign) | 1659 |
| Meningitis | 1247 |
| Motor Neuron Disease | 1259 |
| Neurological injury/trauma | 1240 |
| Spina bifida | 1524 |
| Subdural hematoma | 1083 |
| Subarachnoid hemorrhage | 1086 |
| Transient ischemic attack | 1082 |

**Supplementary Method 2: Ascertainment of heart disease, diabetes, and hypertension**

*Heart disease*

Heart disease was ascertained by the linkage to medical records (Field 131297, ICD I20-I25) or self-reported medical history (Fields 3627, 3894, 6150, and 20002 [1074 and 1075]) including myocardial infarction, heart attack, angina, and myocardial infarction.

*Diabetes*

Participants were classified as having diabetes if they had any of the following: hemoglobin A1c ≥6.5%, fasting plasma glucose ≥126 mg/dl, random blood glucose ≥200 mg/dl, a self-reported history of diabetes (Fields 2443, 2976, and 20002 [1220, 1222, 1223]), use of glucose-lowering medications (Fields 6153 and 6177), or medical record of diabetes (Field 130706, ICD E10-E14).

*Hypertension*

Blood pressure was measured using the Omron 705 IT electronic blood pressure monitor at baseline. Two readings were recorded for each participant and the average of the two measurements was calculated. Hypertension was defined as systolic blood pressure ≥140 mm Hg, diastolic blood pressure ≥90 mm Hg, a self-reported history of hypertension (Fields 2966, 6150, and 20002 [1065 and 1072]), the use of antihypertensive drugs (Fields 6153 and 6177), or medical record of hypertension (Fields 131286-131295, ICD I10-I13 and I15).

**Supplementary Method 3: Details on cognitive reserve-related variables and latent class analysis**

Education, occupational attainment, television viewing time, frequency of confiding, frequency of social connection, and number of leisure activities reflect different aspects of cognitive reserve (CR), and thus we used these six variables to generate an overall CR indicator.

*1) Education level*

Participants were required to indicate whether they possessed one or more of the following qualifications: “college/university degree”, “A levels/AS levels or equivalent”, “O levels/GCSEs or equivalent”, “CSEs or equivalent”, “NVQ or HND or HNC or equivalent”, “Other professional qualifications eg: nursing, teaching”, and “None of the above”. We defined education level based on the maximum years of schooling corresponding to these qualifications [1].

*2) Occupational attainment*

Participants were required to indicate their job titles and employment status. Job titles were coded as 8-digit job codes and presented in a tree structure which were derived from the Standard Occupational Classification 2000 system, developed by the UK Office of National Statistics [2]. A truncated version of the fully detailed job codes was obtained where participants were grouped according to the first 4 digits of their job codes. The socio-economic classification (SEC) in analytic classes was directly derivable from these codes [3]; thus, SEC was an occupationally based classification and reflected occupational attainment. Simplified SEC was rated as the ordinal variables: 1.1 (large employers and higher managerial occupations), 1.2 (higher professional occupations), 2 (lower managerial and professional occupations), 3 (intermediate occupations), 4 (small employers and own account workers), 5 (lower supervisory and technical occupations), 6 (semi-routine occupations), and 7 (routine occupations). Here, we called the simplified SEC to define occupational attainment. An additional category, reflecting the lowest level of occupational attainment, included those who were unemployed, looking after home and/or family, or unable to work because of sickness or disability. If participants only indicated that they were retired, their occupational attainment was treated as missing data.

*3) Television viewing time*

Participants were required to indicate how many hours they spent watching television in a typical day. If the time participant spent on television viewing varied a lot, then the participant was asked to give the average time for a 24-hour day in the last 4 weeks. The following checks were performed: if <0 or >24 the answer was rejected; if >8 then the participant was asked to confirm the answer.

*4) Frequency of confiding in others*

Participants were required to indicate how often they were able to confide in someone close to them, with possible answers consisting of “almost daily”, “2-4 times a week”, “about once a week”, “about once a month”, “once every few months”, or “never or almost never”.

*5) Frequency of social connection*

Participants were required to indicate how often they made or received friend/family visits, with possible answers consisting of “almost daily”, “2-4 times a week”, “about once a week”, “about once a month”, “once every few months”, “never or almost never” or “no friends/family outside household”.

*6) Number of leisure activity types*

Participants were required to indicate which one from a list of activities they attended once a week or more often. These activities consisted of “sports club or gym”, “pub or social club”, “religious group”, “adult education class”, and “other group activity”. Total number of activities selected was regarded as the number of leisure activities. Answers corresponding to “none of above” were coded as “0”.

For the above six questions, answers corresponding to “do not know” or “prefer not to answer” were treated as missing data.

Latent class analysis (LCA) was conducted to create the CR indicator using the SAS command (*PROC LCA*). Likelihood ratio statistic *G*^2^, Akaike information criterion (AIC), and Bayesian information criterion (BIC) were used for model selection, with lower values indicating a more reasonable model. We conduct the models with one to six latent classes, and the statistic parameters are reported in the following table.

**Table 2 for supplementary method.** *G*^2^ statistics, AIC, and BIC in models with different numbers of latent classes

| **Models** | ***G*^2^** | **AIC** | **BIC** |
| --- | --- | --- | --- |
| 1-class model | 18042.65 | 18076.65 | 18223.74 |
| 2-class model | 6405.09 | 6475.09 | 6777.93 |
| 3-class model | 5047.61 | 5153.61 | 5612.19 |
| 4-class model | 4239.78 | 4381.78 | 4996.11 |
| 5-class model | 3853.13 | 4031.13 | 4801.21 |
| 6-class model | 3622.96 | 3836.96 | 4762.79 |

Furthermore, the mean posterior probabilities in models with three to six latent classes were examined to assess the uncertainty of posterior classification, with a value of 0.7 or more indicating an acceptable uncertainty. As shown in the following table, all mean posterior probabilities from the three-latent-class solution were all ≥0.7, and therefore the three-latent-class model was ideal for the uncertainty of posterior classification.

**Table 3 for supplementary method.** Mean posterior probabilities and class membership probabilities in models with three to six latent classes

| **Probabilities** | **Latent class 1** | **Latent class 2** | **Latent class 3** | **Latent class 4** | **Latent class 5** | **Latent class 6** |
| --- | --- | --- | --- | --- | --- | --- |
| **Three-latent-class solution** | | | | | | |
| Mean posterior probabilities | 0.86 | 0.72 | 0.75 | - | - | - |
| Class membership probabilities | 0.37 | 0.27 | 0.36 | - | - | - |
| **Four-latent-class solution** | | | | | | |
| Mean posterior probabilities | 0.61 | 0.68 | 0.79 | 0.65 | - | - |
| Class membership probabilities | 0.20 | 0.26 | 0.26 | 0.27 | - | - |
| **Five-latent-class solution** | | | | | | |
| Mean posterior probabilities | 0.65 | 0.67 | 0.68 | 0.61 | 0.59 | - |
| Class membership probabilities | 0.16 | 0.27 | 0.17 | 0.22 | 0.18 | - |
| **Six-latent-class solution** | | | | | | |
| Mean posterior probabilities | 0.63 | 0.56 | 0.71 | 0.55 | 0.58 | 0.64 |
| Class membership probabilities | 0.15 | 0.17 | 0.16 | 0.14 | 0.16 | 0.22 |

In the three-latent-class model, *Latent class 1* had higher levels of education and occupational attainment and a lower level of time spent watching television; *Latent class 2* had higher levels of frequency of confiding, frequency of social connection, and number of leisure activities. Nevertheless, the overall level of the six CR-related variables in *Latent class 1* was higher than that in *Latent class 2* according to comprehensive item-response probabilities. Additionally, there were overall less favorable levels of CR-related factors in *Latent class 3*. Thus, *Latent class 1*, *Latent class 2*, and *Latent class 3* could be defined as “high CR”, “moderate CR”, and “low CR”, respectively.

References

[1] Rietveld CA, Medland SE, Derringer J, et al. GWAS of 126,559 individuals identifies genetic variants associated with educational attainment. Science 2013;340(6139):1467-1471.

[2] Office for National Statistics. Standard Occupational Classification 2000, Volume 1: Structure and descriptions of unit groups. The Stationery Office; 2000.

[3] Office for National Statistics. The National Statistics Socio-economic Classification User Manual 2005. [https://www.ons.gov.uk/ons/guide-method/classifications/archived-standard-classifications/soc-and-sec-archive/the-national-statistics-socio-economic-classification%2D%2Duser-manual.pdf](%20https:/www.ons.gov.uk/ons/guide-method/classifications/archived-standard-classifications/soc-and-sec-archive/the-national-statistics-socio-economic-classification%2D%2Duser-manual.pdf). Accessed 20 Dec. 2022.

**Appendix 2**

**Supplementary Table 1** Distribution characteristics of levels of cognitive reserve-related variables in three cognitive reserve classes

| **Characteristics** | **No. of subjects** | **Cognitive reserve** | | |
| --- | --- | --- | --- | --- |
|  |  | Low  (n=16032) | Moderate  (n=10709) | High  (n=15560) |
| Education level |  |  |  |  |
| No educational qualifications, CSEs, O levels/GCSE or equivalent | 7919 (18.72) | 7823 (48.80) | 96 (0.90) | 0 |
| A/AS levels or equivalent, other professional qualifications | 7748 (18.32) | 3166 (19.75) | 3112 (29.06) | 1470 (9.45) |
| NVQ, HND, HNC or equivalent | 6485 (15.33) | 5007 (31.32) | 569 (5.31) | 909 (5.84) |
| College/university degree | 20149 (47.64) | 36 (0.22) | 6932 (64.73) | 13181 (84.71) |
| Occupational attainment |  |  |  |  |
| Unemployed or SEC 5–7 | 6260 (14.80) | 4897 (30.55) | 705 (6.58) | 658 (4.23) |
| SEC 4 or SEC 3 | 7982 (18.87) | 5929 (36.98) | 948 (8.85) | 1105 (7.10) |
| SEC 2 | 14302 (33.81) | 3216 (20.06) | 6015 (56.17) | 5071 (32.59) |
| SEC 1.2 or SEC 1.1 | 13757 (32.52) | 1990 (12.41) | 3041 (28.40) | 8726 (56.08) |
| Television viewing time (hours/day) |  |  |  |  |
| ≥4 | 7797 (18.43) | 5168 (32.24) | 1104 (10.31) | 1525 (9.80) |
| 3–3.9 | 9373 (22.16) | 4409 (27.50) | 2110 (19.70) | 2854 (18.34) |
| 2–2.9 | 13285 (31.41) | 4239 (26.44) | 3651 (34.09) | 5395 (34.67) |
| <2 | 11846 (28.00) | 2216 (13.82) | 3844 (35.90) | 5786 (37.19) |
| Frequency of confiding |  |  |  |  |
| Never | 5251 (12.41) | 2603 (16.24) | 132 (1.23) | 2516 (16.17) |
| Less than about once a month | 4520 (10.69) | 1762 (10.99) | 570 (5.32) | 2188 (14.06) |
| About once a week | 4483 (10.60) | 1600 (9.98) | 1302 (12.16) | 1581 (10.16) |
| 2–4 times a week | 4340 (10.26) | 1365 (8.51) | 1929 (18.01) | 1046 (6.72) |
| Almost daily | 23707 (56.04) | 8702 (54.28) | 6776 (63.27) | 8229 (52.89) |
| Frequency of social connection |  |  |  |  |
| Less than about once a month | 9946 (23.51) | 3074 (19.17) | 16 (0.15) | 6856 (44.06) |
| About once a week | 16247 (38.41) | 6130 (38.24) | 2456 (22.93) | 7661 (49.24) |
| More than twice a week | 16108 (38.08) | 6828 (42.59) | 8237 (76.92) | 1043 (6.70) |
| Number of leisure activity types (/week) | | | | |
| ≤0 | 9941 (23.50) | 5419 (33.80) | 1268 (11.84) | 3254 (20.91) |
| 1 | 18106 (42.80) | 7130 (44.47) | 3786 (35.35) | 7190 (46.21) |
| 2–5 | 14254 (33.70) | 3483 (21.73) | 5655 (52.81) | 5116 (32.88) |

Abbreviations: CSE, Certificate of Secondary Education; GCSE, General Certificate of Secondary Education; NVQ, National Vocational Qualification; HND, Higher National Diploma; HNC, Higher National Certificate; SEC, socio-economic classification

**Supplementary Table 2** Baseline characteristics of the subsample with MRI data by different levels of cognitive reserve (n=34,041)

| **Characteristics** | **Cognitive reserve** | | | ***P*** |
| --- | --- | --- | --- | --- |
|  | Low  (n=12636) | Moderate  (n=8723) | High  (n=12682) |  |
| Age | 54.46±7.37 | 54.77±7.40 | 53.36±7.28 | <0.001 |
| Female | 6985 (55.28) | 5443 (62.40) | 5660 (44.63) | <0.001 |
| Ethnicity-White | 11930 (94.41) | 8073 (92.55) | 11424 (90.08) | <0.001 |
| Cigarette smoking |  |  |  | <0.001 |
| Never | 7147 (56.69) | 5585 (64.13) | 8259 (65.16) |  |
| Previous | 4495 (35.65) | 2653 (30.46) | 3744 (29.54) |  |
| Current | 965 (7.65) | 471 (5.41) | 671 (5.29) |  |
| Alcohol consumption |  |  |  | <0.001 |
| Never | 329 (2.60) | 186 (2.13) | 280 (2.21) |  |
| Previous | 301 (2.38) | 150 (1.72) | 241 (1.90) |  |
| Current | 12002 (95.01) | 8387 (96.15) | 12159 (95.89) |  |
| Physical activity |  |  |  | <0.001 |
| Low | 2005 (18.89) | 1080 (14.12) | 2368 (20.72) |  |
| Moderate | 5081 (47.88) | 4361 (57.02) | 6733 (58.91) |  |
| High | 3526 (33.23) | 2207 (28.86) | 2328 (20.37) |  |
| Body mass index (kg/m^2^) | 27.11±4.40 | 26.18±4.10 | 26.10±4.09 | <0.001 |
| Heart disease | 432 (3.42) | 185 (2.21) | 265 (2.09) | <0.001 |
| Diabetes | 396 (3.31) | 171 (1.96) | 292 (2.30) | <0.001 |
| Hypertension | 3034 (24.01) | 1753 (20.10) | 2497 (19.69) | <0.001 |
| Apolipoprotein E ε4 carriers | 2902 (27.59) | 2024 (27.26) | 2993 (27.78) | 0.744 |
| Global cognitive function | 0.03±0.67 | 0.15±0.65 | 0.19±0.66 | <0.001 |
| Numeric memory | 0.00±0.94 | 0.17±0.93 | 0.28±0.91 | <0.001 |
| Prospective memory | 0.03±0.91 | 0.12±0.73 | 0.12±0.74 | <0.001 |
| Pairs matching | -0.08±0.98 | 0.01±1.00 | 0.02±1.02 | <0.001 |
| Fluid intelligence | -0.35±0.91 | 0.20±0.95 | 0.29±0.98 | <0.001 |
| Reaction time | 0.20±0.98 | 0.26±0.95 | 0.34±0.93 | <0.001 |

Data are presented as means ± standard deviations or number (proportion, %).

Missing data: 51 for cigarette smoking; 6 for alcohol consumption; 33 for body mass index; 4,352 for physical activity; 5,324 for apolipoprotein E ε4 status.

**Supplementary Table 3** Standardized betas (β) and 95% confidence intervals (CIs) for the association of cognitive reserve (CR) with changes of cognitive function over the follow-up: results from linear mixed-effects models

| **CR** | **Global cognitive function** | | **Numeric memory** | | **Prospective memory** | |
| --- | --- | --- | --- | --- | --- | --- |
|  | β (95% CI) | *P*-value | β (95% CI) | *P*-value | β (95% CI) | *P*-value |
| **Total sample (n=42301)** |  |  |  |  |  |  |
| Low CR × time | Reference |  | Reference |  | Reference |  |
| Moderate CR × time | 0.06 (0.04, 0.08) | <0.001 | 0.05 (-0.04, 0.14) | 0.263 | 0.08 (0.03, 0.13) | 0.002 |
| High CR × time | 0.10 (0.08, 0.11) | <0.001 | 0.03 (-0.04, 0.11) | 0.462 | 0.10 (0.06, 0.15) | <0.001 |
| **Age stratification** |  |  |  |  |  |  |
| Aged <60 years (n=30218) | |  |  |  |  |  |
| Low CR × time | Reference |  | Reference |  | Reference |  |
| Moderate CR × time | 0.07 (0.04, 0.09) | <0.001 | 0.03 (-0.07, 0.13) | 0.557 | 0.05 (0.01, 0.10) | 0.048 |
| High CR × time | 0.11 (0.09, 0.13) | <0.001 | 0.01 (-0.08, 0.10) | 0.828 | 0.08 (0.03, 0.13) | 0.008 |
| Aged 60+ years (n=12083) | |  |  |  |  |  |
| Low CR × time | Reference |  | Reference |  | Reference |  |
| Moderate CR × time | 0.05 (0.02, 0.08) | 0.001 | 0.21 (0.04, 0.37) | 0.010 | 0.14 (0.04 0.24) | 0.006 |
| High CR × time | 0.07 (0.03, 0.10) | <0.001 | 0.07 (-0.10, 0.24) | 0.420 | 0.13 (0.04, 0.23) | 0.011 |

Models were adjusted for age, sex, ethnicity, cigarette smoking, alcohol consumption, physical activity, body mass index, heart disease, diabetes, hypertension, and apolipoprotein E ε4.**Supplementary Table 3** **(Continued)** Standardized betas (β) and 95% confidence intervals (CIs) for the association of cognitive reserve (CR) with changes of cognitive function over the follow-up: results from linear mixed-effects models

| **CR** | **Pairs matching** | | **Fluid intelligence** | | **Reaction time** | |
| --- | --- | --- | --- | --- | --- | --- |
|  | β (95% CI) | *P*-value | β (95% CI) | *P*-value | β (95% CI) | *P*-value |
| **Total sample (n=42301)** |  |  |  |  |  |  |
| Low CR × time | Reference |  | Reference |  | Reference |  |
| Moderate CR × time | -0.03 (-0.07, 0.01) | 0.326 | 0.01 (-0.03, 0.05) | 0.544 | 0.02 (-0.01, 0.04) | 0.398 |
| High CR × time | -0.02 (-0.05, 0.01) | 0.096 | 0.07 (0.04, 0.10) | <0.001 | 0.04 (0.02, 0.06) | <0.001 |
| **Age stratification** |  |  |  |  |  |  |
| Aged <60 years (n=30218) | |  |  |  |  |  |
| Low CR × time | Reference |  | Reference |  | Reference |  |
| Moderate CR × time | -0.05 (-0.08, 0.002) | 0.061 | 0.01 (-0.04, 0.05) | 0.823 | 0.01 (-0.02, 0.04) | 0.568 |
| High CR × time | -0.03 (-0.06, 0.004) | 0.071 | 0.07 (0.03, 0.11) | <0.001 | 0.05 (0.02, 0.07) | <0.001 |
| Aged 60+ years (n=12083) | |  |  |  |  |  |
| Low CR × time | Reference |  | Reference |  | Reference |  |
| Moderate CR × time | -0.02 (-0.08, 0.04) | 0.258 | 0.02 (-0.03, 0.07) | 0.433 | 0.01 (-0.04. 0.05) | 0.624 |
| High CR × time | -0.02 (-0.08, 0.04) | 0.280 | 0.04 (-0.02, 0.09) | 0.117 | 0.01 (-0.03, 0.06) | 0.549 |

Models were adjusted for age, sex, ethnicity, cigarette smoking, alcohol consumption, physical activity, body mass index, heart disease, diabetes, hypertension, and apolipoprotein E ε4.

**Supplementary Table 4** Standardized betas (β) and 95% confidence intervals (CIs) for the association of cognitive reserve (CR) with changes of cognitive function over the follow-up, stratified by apolipoprotein E ε4 status: results from linear mixed-effects models

| **Cognitive reserve** | **Global cognitive function** | **Numeric memory** | **Prospective memory** | **Pairs matching** | **Fluid intelligence** | **Reaction time** |
| --- | --- | --- | --- | --- | --- | --- |
|  | β (95% CI) | β (95% CI) | β (95% CI) | β (95% CI) | β (95% CI) | β (95% CI) |
| **Carriers of apolipoprotein E ε4** | | | | | | |
| Low CR × time | Reference | Reference | Reference | Reference | Reference | Reference |
| Moderate CR × time | 0.04 (0.01, 0.08) | -0.01 (-0.21, 0.18) | 0.11 (0.02, 0.20) | -0.06 (-0.12, 0.01) | -0.04 (-0.12, 0.04) | -0.01 (-0.06, 0.04) |
| High CR × time | 0.10 (0.07, 0.14) | 0.03 (-0.14, 0.21) | 0.11 (0.02, 0.19) | -0.04 (-0.10, 0.02) | 0.05 (0.02, 0.12) | 0.05 (0.01, 0.09) |
| **Non-carriers of apolipoprotein E ε4** | | | | | | |
| Low CR × time | Reference | Reference | Reference | Reference | Reference | Reference |
| Moderate CR × time | 0.07 (0.05, 0.10) | 0.11 (-0.01, 0.22) | 0.06 (0.01, 0.12) | -0.03 (-0.07, 0.01) | 0.02 (-0.03, 0.07) | 0.02 (-0.01, 0.06) |
| High CR × time | 0.10 (0.08, 0.13) | 0.03 (-0.07, 0.13) | 0.10 (0.04, 0.16) | -0.02 (-0.06, 0.02) | 0.08 (0.03, 0.12) | 0.06 (0.03, 0.08) |

Models were adjusted for age, sex, ethnicity, cigarette smoking, alcohol consumption, physical activity, body mass index, heart disease, diabetes, and hypertension.

**Supplementary Table 5** Least-squares means and 95% confidence intervals (CIs) of brain structural magnetic resonance imaging parameters related to CR: results from analysis of covariance models

| **CR** | **Total brain volumes (mm^3^)** | | | **White matter volumes (mm^3^)** | | | **Grey matter volumes (mm^3^)** | | |
| --- | --- | --- | --- | --- | --- | --- | --- | --- | --- |
|  | Least-squares means  (95% CI) | Least-squares mean differences (95% CI) | *P*-value | Least-squares means (95% CI) | Least-squares mean differences (95% CI) | *P*-value | Least-squares means (95% CI) | Least-squares mean differences (95% CI) | *P*-value |
| **Total sample (n=34041)** | |  |  |  |  |  |  |  |  |
| Low CR | 1167071 (1166253, 1167890) | Reference |  | 548589 (548051, 549127) | Reference |  | 618483 (618018, 618947) | Reference |  |
| Moderate CR | 1164276 (1163302, 1165250) | -2795 (-4240, -1351) | <0.001 | 546437 (545797, 547077) | -2152 (-3101, -1203) | <0.001 | 617839 (617287, 618392) | -643 (-1463, 176) | 0.147 |
| High CR | 1163849 (1163032, 1164666) | -3222 (-4553, -1892) | <0.001 | 546646 (546109, 547183) | -1943 (-2817, -1068) | <0.001 | 617203 (616739, 617666) | -1280 (-2034, -525) | <0.001 |
| **Age stratification** | |  |  |  |  |  |  |  |  |
| Aged <60 years (n=24781) | |  |  |  |  |  |  |  |  |
| Low CR | 1178274 (1177297, 1179252) | Reference |  | 551335 (550693, 551977) | Reference |  | 627130 (626583, 627678) | Reference |  |
| Moderate CR | 1175132 (1173963, 1176301) | -3142 (-4872, -1413) | <0.001 | 548449 (547682, 549216) | -2886 (-4020, -1751) | <0.001 | 626115 (625460, 626769) | -1015 (-1984, -47) | 0.037 |
| High CR | 1175129 (1174197, 1176060) | -3145 (-4698, -1593) | <0.001 | 549413 (548801, 550025) | -1922 (-2942, -902) | <0.001 | 625895 (625374, 626417) | -1235 (-2104, -365) | 0.003 |
| Aged 60+ years (n=9260) | |  |  |  |  |  |  |  |  |
| Low CR | 1137515 (1135989, 1139041) | Reference |  | 541919 (540877, 542960) | Reference |  | 595388 (594515, 596261) | Reference |  |
| Moderate CR | 1134695 (1132899, 1136492) | -2820 (-5503, -137) | 0.038 | 539485 (538233, 540684) | -2460 (-4291, -630) | 0.006 | 595690 (594662, 596718) | 302 (-1233, 1837) | 0.875 |
| High CR | 1133434 (1131702, 1135165) | -4081 (-6736, -1427) | 0.001 | 539560 (538379, 540742) | -2359 (-4170, -547) | 0.008 | 593728 (592738, 594718) | -1660 (-3178, -142) | 0.029 |

Models were adjusted for age, sex, ethnicity, cigarette smoking, alcohol consumption, physical activity, body mass index, heart disease, diabetes, hypertension, and head size, position magnetic resonance imaging confounds, and baseline level of global cognitive function.

**Supplementary Table 5 (Continued)** Least-squares means and 95% confidence intervals (CIs) of brain structural magnetic resonance imaging parameters related to CR: results from analysis of covariance models

| **CR** | **Hippocampal volumes (mm^3^)** | | | **log(White matter hyperintensity volumes) (log(mm^3^))** | | |
| --- | --- | --- | --- | --- | --- | --- |
|  | Least-squares means  (95% CI) | Least-squares mean differences (95% CI) | *P*-value | Least-squares means  (95% CI) | Least-squares mean differences (95% CI) | *P*-value |
| **Total sample (n=34041)** | |  |  |  |  |  |
| Low CR | 7689 (7675, 7703) | Reference |  | 7.96 (7.94, 7.97) | Reference |  |
| Moderate CR | 7701 (7684, 7717) | 12 (-12, 36) | 0.459 | 7.94 (7.93, 7.96) | -0.02 (-0.04, 0.10) | 0.316 |
| High CR | 7723 (7710, 7737) | 35 (12, 57) | 0.001 | 7.91 (7.89, 7.93) | -0.05 (-0.07, -0.02) | <0.001 |
| **Age stratification** | |  |  |  |  |  |
| Aged <60 years (n=24781) | |  |  |  |  |  |
| Low CR | 7803 (7787, 7819) | Reference |  | 7.74 (7.72, 7.75) | Reference |  |
| Moderate CR | 7818 (7799, 7837) | 15 (-13, 44) | 0.385 | 7.72 (7.70, 7.74) | -0.02 (-0.05, 0.01) | 0.294 |
| High CR | 7845 (7830, 7860) | 42 (18, 68) | <0.001 | 7.69 (7.67, 7.71) | -0.05 (-0.07, -0.02) | <0.001 |
| Aged 60+ years (n=9260) | |  |  |  |  |  |
| Low CR | 7383 (7357, 7409) | Reference |  | 8.55 (8.53, 8.58) | Reference |  |
| Moderate CR | 7388 (7358, 7419) | 5 (-40, 51) | 0.956 | 8.55 (8.52, 8.58) | -0.005 (-0.05, 0.04) | 0.959 |
| High CR | 7393 (7363, 7422) | 10 (-35, 55) | 0.856 | 8.51 (8.48, 8.54) | -0.05 (-0.09, 0.003) | 0.069 |

Models were adjusted for age, sex, ethnicity, cigarette smoking, alcohol consumption, physical activity, body mass index, heart disease, diabetes, hypertension, and head size, position magnetic resonance imaging confounds, and baseline level of global cognitive function.

**Supplementary Table 6** Standardized betas (β) and 95% confidence intervals (CIs) for the association of cognitive reserve (CR) with changes of global cognitive function over the follow-up: results from linear mixed-effects models

| **Cognitive reserve** | **Global cognitive function** | | | | |
| --- | --- | --- | --- | --- | --- |
|  | β (95% CI)^a^ | β (95% CI)^b^ | β (95% CI)^c^ | β (95% CI)^d^ | β (95% CI)^e^ |
| Low CR × time | Reference | Reference | Reference | Reference | Reference |
| Moderate CR × time | 0.06 (0.04, 0.08) | 0.06 (0.04, 0.08) | 0.06 (0.04, 0.08) | 0.06 (0.04, 0.08) | 0.06 (0.04, 0.08) |
| High CR × time | 0.09 (0.08, 0.11) | 0.09 (0.08, 0.11) | 0.09 (0.08, 0.11) | 0.09 (0.08, 0.11) | 0.09 (0.08, 0.11) |

Models were adjusted for age, sex, ethnicity, cigarette smoking, alcohol consumption, physical activity, body mass index, heart disease, diabetes, hypertension, and apolipoprotein E ε4.

Models were further adjusted ^a^total brain volume, ^b^white matter volume, ^c^grey matter volume, ^d^hippocampal volume, and ^e^white matter hyperintensity volume.**Supplementary Table 7** Standardized betas (β) and 95% confidence intervals (CIs) for the association of cognitive reserve (CR) with changes of global cognitive function over the follow-up, stratified by regional brain volume: results from linear mixed-effects models

| **Cognitive reserve** | **Global cognitive function** | | | | |
| --- | --- | --- | --- | --- | --- |
|  | β (95% CI) | β (95% CI) | β (95% CI) | β (95% CI) | β (95% CI) |
|  | **Smaller total brain** | **Smaller white matter** | **Smaller grey matter** | **Smaller hippocampus** | **Larger WMH** |
| Low CR × time | Reference | Reference | Reference | Reference | Reference |
| Moderate CR × time | 0.06 (0.03, 0.09) | 0.07 (0.04, 0.10) | 0.07 (0.04, 0.10) | 0.07 (0.04, 0.10) | 0.07 (0.04, 0.10) |
| High CR × time | 0.10 (0.07, 0.12) | 0.10 (0.07, 0.12) | 0.09 (0.07, 0.12) | 0.10 (0.08, 0.13) | 0.09 (0.06, 0.12) |
|  | **Larger total brain** | **Larger white matter** | **Larger grey matter** | **Larger hippocampus** | **Smaller WMH** |
| Low CR × time | Reference | Reference | Reference | Reference | Reference |
| Moderate CR × time | 0.08 (0.05, 0.10) | 0.07 (0.04, 0.10) | 0.07 (0.04, 0.10) | 0.07 (0.04, 0.10) | 0.06 (0.03, 0.09) |
| High CR × time | 0.10 (0.08, 0.13) | 0.10 (0.07, 0.13) | 0.10 (0.08, 0.13) | 0.09 (0.07, 0.12) | 0.10 (0.08, 0.13) |
| *P*-interaction for moderate CR | 0.531 | 0.940 | 0.962 | 0.906 | 0.724 |
| *P-*interaction for high CR | 0.850 | 0.891 | 0.608 | 0.681 | 0.549 |

Models were adjusted for age, sex, ethnicity, cigarette smoking, alcohol consumption, physical activity, body mass index, heart disease, diabetes, hypertension, and apolipoprotein E ε4.

Abbreviations: WMH, white matter hyperintensity

**Supplementary Table 8** Standardized betas (β) and 95% confidence intervals (CIs) for the association of cognitive reserve (CR) with changes of cognitive function over the follow-up among the MRI subsample: results from linear mixed-effects models

| **Cognitive reserve** | **Global cognitive function** | **Numeric memory** | **Prospective memory** | **Pairs matching** | **Fluid intelligence** | **Reaction time** |
| --- | --- | --- | --- | --- | --- | --- |
|  | β (95% CI) | β (95% CI) | β (95% CI) | β (95% CI) | β (95% CI) | β (95% CI) |
| Low CR × time | Reference | Reference | Reference | Reference | Reference | Reference |
| Moderate CR × time | 0.07 (0.05, 0.08) | 0.07 (-0.02, 0.16) | 0.09 (0.04, 0.14) | -0.04 (-0.08, 0.01) | 0.02 (-0.02, 0.06) | 0.02 (-0.01, 0.03) |
| High CR × time | 0.10 (0.08, 0.12) | 0.02 (-0.06, 0.10) | 0.09 (0.05, 0.14) | -0.03 (-0.07, 0.01) | 0.06 (0.03, 0.10) | 0.05 (0.02, 0.07) |

Models were adjusted for age, sex, ethnicity, cigarette smoking, alcohol consumption, physical activity, body mass index, heart disease, diabetes, hypertension, and apolipoprotein E ε4.

**Supplementary Table 9** Standardized betas (β) and 95% confidence intervals (CIs) for the association of cognitive reserve (CR) with changes of cognitive function over the follow-up by excluding incident dementia cases: results from linear mixed-effects models

| **Cognitive reserve** | **Global cognitive function** | **Numeric memory** | **Prospective memory** | **Pairs matching** | **Fluid intelligence** | **Reaction time** |
| --- | --- | --- | --- | --- | --- | --- |
|  | β (95% CI) | β (95% CI) | β (95% CI) | β (95% CI) | β (95% CI) | β (95% CI) |
| Low CR × time | Reference | Reference | Reference | Reference | Reference | Reference |
| Moderate CR × time | 0.06 (0.05, 0.08) | 0.08 (-0.01, 0.16) | 0.08 (0.04, 0.12) | -0.04 (-0.07, 0.01) | -0.01 (-0.04, 0.03) | 0.02 (-0.01, 0.04) |
| High CR × time | 0.10 (0.08, 0.12) | 0.04 (-0.04, 0.12) | 0.10 (0.06, 0.14) | -0.02 (-0.05, 0.003) | 0.07 (0.04, 0.10) | 0.06 (0.04, 0.08) |

Models were adjusted for age, sex, ethnicity, cigarette smoking, alcohol consumption, physical activity, body mass index, heart disease, diabetes, hypertension, and apolipoprotein E ε4.

**Supplementary Table 10** Standardized betas (β) and 95% confidence intervals (CIs) for the association of cognitive reserve (CR) with changes of cognitive function over the follow-up: results from linear mixed-effects models

| **Cognitive reserve** | **Global cognitive function** | **Numeric memory** | **Prospective memory** | **Pairs matching** | **Fluid intelligence** | **Reaction time** |
| --- | --- | --- | --- | --- | --- | --- |
|  | β (95% CI) | β (95% CI) | β (95% CI) | β (95% CI) | β (95% CI) | β (95% CI) |
| Low CR × time | Reference | Reference | Reference | Reference | Reference | Reference |
| Moderate CR × time | 0.06 (0.04, 0.08) | 0.05 (-0.04, 0.14) | 0.08 (0.03, 0.12) | -0.03 (-0.07, 0.01) | 0.01 (-0.03, 0.05) | 0.02 (-0.01, 0.04) |
| High CR × time | 0.10 (0.08, 0.12) | 0.03 (-0.04, 0.11) | 0.10 (0.06, 0.15) | -0.02 (-0.05, 0.003) | 0.07 (0.04, 0.10) | 0.04 (0.02, 0.06) |

Models were adjusted for age, sex, ethnicity, cigarette smoking, alcohol consumption, physical activity, body mass index, heart disease, diabetes, hypertension, apolipoprotein E ε4, and the Townsend deprivation index.

**Supplementary Table 11** Standardized betas (β) and 95% confidence intervals (CIs) for the association of cognitive reserve (CR) with changes of cognitive function over the follow-up, stratified by sex: results from linear mixed-effects models

| **Cognitive reserve** | **Global cognitive function** | **Numeric memory** | **Prospective memory** | **Pairs matching** | **Fluid intelligence** | **Reaction time** |
| --- | --- | --- | --- | --- | --- | --- |
|  | β (95% CI) | β (95% CI) | β (95% CI) | β (95% CI) | β (95% CI) | β (95% CI) |
| **Women** | | | | | | |
| Low CR × time | Reference | Reference | Reference | Reference | Reference | Reference |
| Moderate CR × time | 0.06 (0.04, 0.09) | 0.03 (-0.10, 0.16) | 0.05 (-0.01, 0.12) | -0.04 (-0.08, 0.01) | 0.01 (-0.04, 0.05) | 0.02 (-0.02, 0.05) |
| High CR × time | 0.11 (0.08, 0.13) | 0.03 (-0.10, 0.15) | 0.11 (0.04, 0.17) | -0.01 (-0.05, 0.04) | 0.08 (0.03, 0.13) | 0.05 (0.02, 0.08) |
| **Men** | | | | | | |
| Low CR × time | Reference | Reference | Reference | Reference | Reference | Reference |
| Moderate CR × time | 0.05 (0.02, 0.08) | 0.08 (-0.06, 0.21) | 0.12 (0.04, 0.20) | -0.03 (-0.07, 0.01) | 0.02 (-0.04, 0.08) | 0.01 (-0.03, 0.04) |
| High CR × time | 0.09 (0.07, 0.12) | -0.003 (-0.11, 0.11) | 0.11 (0.04, 0.17) | -0.04 (-0.09, 0.01) | 0.07 (0.02, 0.12) | 0.03 (0.001, 0.06) |

Models were adjusted for age, ethnicity, cigarette smoking, alcohol consumption, physical activity, body mass index, heart disease, diabetes, hypertension, and apolipoprotein E ε4.
